# Should digestion assays be used to estimate persistence of potential allergens in tests for safety of novel food proteins?

**DOI:** 10.1186/1476-7961-7-1

**Published:** 2009-01-15

**Authors:** Santiago Schnell, Rod A Herman

**Affiliations:** 1 Department of Molecular & Integrative Physiology, and Center for Computational Medicine & Biology, University of Michigan Medical School, 100 Washtenaw Avenue, Palmer Commons 2017, Ann Arbor, MI 48109-2218, USA; 2 Dow AgroSciences LLC, 9330 Zionsville Rd., Indianapolis, IN 46268, USA

## Abstract

Food allergies affect an estimated 3 to 4% of adults and up to 8% of children in developed western countries. Results from *in vitro *simulated gastric digestion studies with purified proteins are routinely used to assess the allergenic potential of novel food proteins. The digestion of purified proteins in simulated gastric fluid typically progresses in an exponential fashion allowing persistence to be quantified using pseudo-first-order rate constants or half lives. However, the persistence of purified proteins in simulated gastric fluid is a poor predictor of the allergenic status of food proteins, potentially due to food matrix effects that can be significant *in vivo*. The evaluation of the persistence of novel proteins in whole, prepared food exposed to simulated gastric fluid may provide a more correlative result, but such assays should be thoroughly validated to demonstrate a predictive capacity before they are accepted to predict the allergenic potential of novel food proteins.

## Background

The adult human gastrointestinal tract (GI) is a tube approximately 9 meters long, running through the body from the mouth to the anus. The lumen of the GI tract is continuous with the external environment, keeping its contents outside of the rest of the body. The epithelial layer, which lines the interior of the GI tract, presents a partial barrier to invasion by ingested pathogens, parasites, toxins and antinutrients. If pathogens, toxins and food proteins breach the epithelium barrier, the immune system acts as our primary defense system. Antibodies are formed that specifically react with epitopes on certain antigenic proteins, and subsequent binding of subtypes of these antibodies to proteins can result in the mobilization of host defenses, including deleterious responses like allergy.

The GI tract helps prevent food antigen penetration through its gut epithelial barrier. Epithelial cells are joined together with their neighbors via tight junctions and mucus produced by goblet cells [1]. In the upper bowel, the bulk of antigen exposure comes from foods, while in the lower bowel, the antigenic load comes from the complex microflora living in the GI tract. In addition to serving as a barrier, the mucosal system has two robust adaptive immune mechanisms to prevent general antigen circulation: (i) antigen exclusion mediated through the secretion of IgA and IgM antibodies to modulate the colonization of microorganisms and dampen penetration of soluble luminal agents, and (ii) suppressive mechanisms to avoid hypersensitivity to substances present in the mucosal surface [2]. The latter mechanism is known as oral tolerance when it is induced by food antigens [3].

Despite these host defense mechanisms, antigens can be absorbed and distributed in the body. Intact food proteins can be detected in plasma [4-6] and gut bacteria can be detected in mesenteric lymph nodes [7]. An estimated 3 to 4% of adults and up to 8% of children suffer from food allergies in developed western countries [8,9]. In the western world, most infectious diseases of the gut are largely under control, yet food allergies are considered to be a major health concern. Food allergy accounts for up to 50% of anaphylactic episodes resulting in hospitalizations [10,11].

Failure of oral tolerance leading to food allergies is most often due to an IgE-mediated hypersensitivity to a small subset of proteins found in milk, eggs, peanuts, fish, shellfish, soy, wheat and tree nuts [12]. Typical diets contain tens of thousands of different proteins, and efforts to understand the unique physiochemical and molecular properties of food allergens are ongoing [13-15].

The exact site of food absorption and allergy induction is still unknown. It is believed that most food allergens are absorbed in the intestines, prior to initiating an immune response, requiring proteins to move through the stomach in an immunologically intact form. Food protein can also enter the circulation through the oral mucosa [16,17]. Certain disease conditions, such as celiac disease, can increase the amount of intact proteins in general circulation [18].

The majority of ingested food proteins break down as they travel through the GI tract. This occurs through the processes of digestion, where the food is exposed to the denaturing environment of hydrochloric acid in the stomach, bile from the liver and digestive enzymes released by the salivary glands, chief cells in the stomach, and the pancreas. The proteases and peptidases produced and secreted by chief cells and the pancreas digest proteins into small peptides typically less than 8 amino acids in size [19]. This extensive digestion renders these peptides non-reactive for antigen recognition [20]. For this reason, resistance to proteolysis has been considered a promising indicator of allergenic potential [21]. More recently Utersmayr and Jensen-Jarolim [22] have shown that antiulcer agents increase the risk of food allergy by interfering with the digestive function and decreasing the threshold of allergens required to elicit symptoms in patients with food allergy. Therefore, when the gastric digestion of a protein is impaired or limited, protein persistence increases, potentially triggering sensitization or allergic symptoms. This phenomenon is known as allergen persistence [22].

Based on the relationship between GI digestion and food allergy, results of *in vitro *digestion experiments have been considered to assess the allergenic potential of new food proteins. In this paper, we review the influence of gastric digestion on the development of food allergy, and evaluate the currently applied digestion assays for testing the allergenic potential of novel food proteins. We start by defining a food allergen, and then discuss the standard simulated gastric fluid digestion (SGF) assay currently used to assess allergenic potential of food proteins. We found that results from SGF assays with pure proteins are not a good predictor of the allergenic potential of food proteins, but rather that they simply measure the resistance of purified food proteins to *in vitro *digestion. Moreover resistance to SGF is not a sufficient or useful criterion for evaluating food allergen sensitization or induction.

### What is a food allergen?

Before we discuss the use of digestion experiments for predicting the allergenic potential of food proteins, we must define a "food allergen". This term is general and ambiguous. Food allergens have at least three potential attributes:

(1) Induction of allergic sensitization.

(2) Reaction with IgE antibodies

(3) Induction of allergic reactions.

The food proteins which do all three of the above are known as complete food allergens [23], while the others are called incomplete food allergens. Incomplete food allergens are divided into two categories [24]: (i) non-elicitors, which do (2), but not (1) or (3), and (ii) non-sensitizing elicitors, which do (2) and (3), but not (1). Bannon [25] suggests that complete allergens are resistant to digestion in the GI tract, while incomplete allergens are potentially susceptible to digestion in the GI tract [26,27].

### The standard digestion assay to assess allergenic potential of food proteins

Digestion assays in simulated gastric fluid (SGF) are commonly employed to predict the allergenic potential of food proteins [28-31], and are currently required as part of the allergenicity assessment of transgenic proteins expressed in food crops [32,33]. Astwood et al. [34] used the SGF assays to investigate the stability of 25 food proteins to pepsin. The hypothesis was that food allergens would survive the acidic gastric environment and resist digestion by pepsin in the stomach to reach the intestinal mucosa and be absorbed, while non-allergens would not [35]. Astwood et al. [34] found that the stability to digestion is significant in the selected food allergens, and concluded that digestion is a valid parameter that distinguishes food allergens from non-allergens.

#### The simulated gastric fluid assay

As a result of the Astwood et al. [34] report, the SGF assay has been incorporated in the decision tree or weight-of-evidence approach to evaluate the allergenic potential of novel food proteins that may be present in food crops [32,33]. The SGF assay has been standardized to facilitate comparisons among substrates [36]. This recipe specifies 0.32% pepsin in hydrochloric acid at a pH of 1.2. SGF was developed to provide a model system for mammalian monogastric digestion and has been used to evaluate the relative nutritional value of different protein sources, and the dissolution of pharmaceuticals [37,38]. It is widely understood that the SGF assay does not actually replicate the gastric environment but only represents a standardized model system for proteolysis under acidic conditions. The SGF assay was first used to systematically evaluate the gastric stability of allergenic food proteins by Astwood et al. [34]. In this study, 0.017% protein substrate was incubated in SGF (0.32% pepsin, pH 1.2) at 37°C.

Pepsin is an aspartic protease generated from the auto-cleavage of pepsinogen under the acidic conditions in the stomach. Pepsin has broad substrate specificity, preferentially cleaving proteins at leucine, phenylalanine and tyrosine [39]. Pepsinolysis is generally very rapid unless hindered by the secondary or tertiary structure of the protein substrate [40-42]. The optimum pH for pepsinolysis is between 1.8 and 3.2, and pepsin is irreversibly denatured at pH 6 to 7 [39,43]. This latter property of pepsin allows the SGF reaction to be stopped by neutralizing aliquots of the solution after different incubation periods. These aliquots can then be analyzed to track the digestion of substrate proteins.

The analytical tool generally used to track the digestion of substrate protein in SGF is sodium dodecyl polyacrylimide gel electrophoresis (SDS-PAGE). SDS-PAGE separates denatured proteins on polyacrylamide gels based primarily on molecular mass, and thus does not distinguish enzyme-bound from non-bound substrate. Proteins are visualized by staining with various dyes such as colloidal Coomassie brilliant blue. While the density of stained bands is generally directly proportional to the protein concentration for any given protein [31,44,45], different proteins have different propensities to bind stain [46]. Thus, the relative concentration of any given protein can be tracked through time, but comparisons of concentration across different proteins are not accurate based solely on band densities. It also follows that the minimum concentration that can be visualized on SDS-PAGE gels differs among different proteins. An example of the dramatic difference in protein staining between two proteins can be seen in Figure 3 in Thomas et al. [38]. In panel B of this figure, the pepsin to ovalbumin ratio is 3:1 w/w, however the ovalbumin band at time zero, prior to digestion, is much darker than the pepsin band.

In some cases, discrete smaller-molecular-weight protein fragments appear, and sometimes disappear, as digestion progresses [38,47]. These digestion fragments may be capable of eliciting an allergic reaction if they have at least two IgE binding sites (epitopes) and are of sufficient size (> 3 kDa) such that the antibody-protein complex can cross-link two receptors on the surface of mast cells causing the cascade of effects leading to an allergic reaction [48]. It is noteworthy that when fragments are seen, they universally appear as discrete bands rather than as smears of many different molecular-weight peptides, indicating that specific fragments likely retain some level of secondary and/or tertiary structure that hinders pepsinolysis.

#### Patterns of digestion in the simulated gastric fluid assays

The SGF assays can produce complex patterns of digestions in SDS-PAGE gels. These patterns revolve around the multiple cleavage sites on the protein substrate rather than from the presence of multiple enzymes or compartments. However, the digestion of the substrate protein generally follows an exponential decline.

The SGF assay is similar to other dissipation experiments, which are conducted to track the disappearance of substrates in complex systems. One example is the tracking of pest-control substances in soil. Microbial digestion of compounds, via many enzymes, in soil often predominates in such systems, and in spite of the complexity of the processes, dissipation of substrate often closely follows a negative exponential pattern [49,50]. Similarly, the clearance of pharmaceuticals from blood also is the result of complex processes often including enzyme catalyzed cleavage, but still generally follows an exponential decline pattern [51]. This same pattern has been observed in a number of *in vitro *protein-protease systems [52], particularly in proteolysis assays under acid-denaturing conditions [53] and pepsinolysis [42,54]. The exponential decay pattern is sometimes biphasic but the final phase of digestion most often follows pseudo-first order kinetics [55]. The progress of the digestion seems to be quite insensitive to variation in both the pepsin concentration and the substrate protein concentration as long as the pepsin concentration is close to that specified in the USP (0.32%), and the substrate protein concentration is relatively low [31,47,56,57].

There are four possible explanations for the biphasic and pseudo-first order decay pattern observed in proteolysis experiments: (i) Protein digestion is dominated by a first-order rate-limiting step. A possible rate-limiting step can be the acid-induced unfolding of the protein under the low pH (1.2) of SGF [42,58]. Unfolding rates have often been found to be critical in proteolysis, and once unfolding occurs, pepsinolysis can proceed very quickly. This would result in apparent exponential disappearance of protein substrate in SGF. (ii) Protein digestion follows pseudo-first-order kinetics [59] under the excess of the digestive enzyme. This is the theory generally used to explain the first-order behavior of protein digestion in SGF [45,52,56,57,60,61]. (iii) In protein digestion assays there is an exponential decay, which is only applicable to the slow transient of the digestion reaction at high enzyme concentrations. Schnell and Maini [62] and Tzafriri [63] have shown that enzyme catalyzed reactions can be described by a first-order kinetics after the initial transient of the reaction at high enzyme concentrations. (iv) The aggregate behavior of complex reactions, such as protein digestion, produces a behavior indistinguishable from the first-order kinetics [64]. Recent computational models have shown that the later theory (iv) provides a compelling explanation for the exponential decay in protein digestion assays [55].

### Is it appropriate to assess the allergenic potential using digestion assays?

While the predictive power of the SGF assay has been promulgated in a number of papers [28-31], and is required as part of the allergenicity assessment of transgenic proteins expressed in food crops [32,33], the predictive power of the assay remains uncertain [47,54,65,66]. Using simulated SGF assays [36], Astwood et al. [34] originally found a good correlation between allergenic status and susceptibility to pepsin under acidic conditions. It was this work that initially prompted the use of the SGF assay to predict the allergenic potential of novel food proteins. However, Fu et al. [65] noticed a confounding factor in the Atwood et al. study. The cellular functions of the proteins evaluated in this investigation were correlated with the allergenic status of the proteins. When a group of allergens and non-allergens were chosen by the latter researchers that controlled for cellular function, the correlation was absent. More recently, Herman et al. [47] found no correlation between the digestibility and allergenic status of seven allergens and eight non-allergens.

Likely reasons for the poor predictive capability of this assay include a lack of consideration of the prevalence of the allergen in food, effects of food processing, and food-matrix interactions [67-73]. The latter factor may be very important since components of food may sequester certain proteins away from the acid and pepsin in gastric fluid. For example, Polovic et al. [73] found that the purified kiwi allergen, Act c 2, was digested quickly in SGF, but was protected from digestion by fruit pectin both *in vitro *and *in vivo*. Similarly, Chikwamba et al. [67] found that transgenic corn expressing the *Escherichia coli *heat-labile enterotoxin facilitated the association of this protein with starch granules that protected it against digestion in SGF. Thus the evaluation of purified proteins in the SGF assay may be misleading.

Also there are a number of complete or potent allergens which are not stable in SGF assays [65,66,74], but their peptide fragments are recognizable by allergen-specific T cells [75]. Digestion outcomes can be influenced by the concentration of substrate protein or pepsin, pH and other factors [76]. Protein allergens of food sources like milk [77], fish [17,78] and hazelnut [75] can be digested *in vitro*, unless the digestion process is inhibited by antacid medication [22]. In the later case, there is an increased risk of food allergy. The sudden increase of food allergy by inhibiting digestion suggests that the concentration of allergens reaching the intestinal mucosa is important in triggering an allergic reaction [79]. A similar phenomenon is observed with gastro-intestinal inflammation diseases, which can increase gut-permeability prior to food allergen contact [7]. This does not imply that allergens are more likely to be stable to digestion in simulated gastric fluid compared with non-allergens, but rather it suggests that if the concentration of a food allergen increases, then the chance of protein absorption is also higher. Once food allergens permeate the GI tract, they will stimulate the immune system to produce IgE antibodies, and degranulate mast cells upon subsequent contact leading to an allergic reaction.

Food allergies are complex, and can be the result of complex interactions. There are also food allergens which can only cause symptoms under cross-reactivity conditions. For example, pollen-allergic patients frequently present food allergies after the ingestion of several plant foods [24]. On the other hand, the mechanisms of how some patients with IgE to ovalbumin tolerate eggs, while others do not, remains unclear [23]. Digestion assays can neither predict the effects of cross-reactivity between food allergens and other antigens, nor the allergic response of a patient to food protein [80].

## Conclusion

Although the value of comparing the stability of proteins in SGF for the purpose of evaluating the allergenic potential of novel food proteins is dubious, such comparisons are routinely used for this purpose. The nature of allergy to food proteins is still unknown. At the moment, we know that the resistance to *in vivo *digestion of an allergenic food protein increases its potential for causing an allergic reaction in susceptible individuals. We also know that some peptide fragments of digested proteins can be recognizable by allergen-specific T cells. However, the amount of food protein and the condition under which can trigger the allergic reaction are largely unknown [81].

### Re-evaluating the application of simulated gastric fluid assay to test food proteins

The limitations of the SGF assays for predicting the allergenic potential are becoming apparent to the food allergy community [47,54,65,66,74]. In light of the limitations of the SGF assays, Utersmayr and Jensen-Jarolim [22] suggested the introduction of a new concept in the food allergen community: allergen persistence. Slow or impaired digestion of food proteins which are potential allergens increases the risk for food allergy induction in sensitized individuals. Although SGF assays with purified proteins cannot predict allergenic potential, they can quantitatively estimate the food protein persistence in the GI tract if food-matrix effects are not significant. If a novel food protein is an allergen, then a dose increase in the GI tract can exceed the threshold for triggering an allergic reaction in sensitized individuals. The typical protein absorption time correlates with gastric transit time determined for pharmaceutical compounds [82].

A kinetic approach to measuring SGF digestion is currently the most reasonable method to quantitatively compare the persistence of purified food proteins during *in vitro *digestion [42,45,47,54,56]. The digestion of proteins in SGF typically conforms to a negative-exponential model allowing first-order rate constants or half lives to characterize the disappearance of substrates over their dissipation profile. This approach provides an *in vitro *measure of the persistence of food proteins.

Apart from the quantitative estimates of protein persistence, other aspects of the SGF assay protocol can also be improved. The evaluation of the persistence of novel proteins in whole, prepared food exposed to SGF [83] may provide better estimates of *in vivo *persistence of food proteins. The proteolysis of food proteins can be affected as a result of processing and interaction with food ingredients. For example, β-lactoglobulin proteolysis by trypsin and chymotrypsin is reduced in the presence of polysaccharides such as gum arabic, low methylated pectin or xylan [84]. Peanut protein digestibility is also reduced in the presence of gum Arabic and xylan [85]. Finally new assays have been proposed to model more realistically the multi-phase nature of the digestive processes [75,84,86]. These digestion assays mimic the passage of the food into the stomach and then into the gut. The development of these digestion assays has demonstrated the importance of using physiologically relevant conditions to investigate the digestion of food proteins *in vitro *[69]. Some of these models have been recently reviewed in [76].

We emphasize that the persistence to SGF *in vitro *provides little value in the absence of evidence that a particular protein can induce IgE antibodies or elicit an allergic response. The allergenic potential of a food can only be diagnosed through sensitive analytical methods which recognize the presence of allergenic antigens in food. For novel food proteins, where populations of allergic individuals are absent or limited, results from SGF assays with pure proteins are of little value in predicting allergenicity. Continued work on new animal models of sensitization for food proteins will be of critical importance for accurately predicting the allergenicity of novel food proteins [87]. SGF assays should be employed for estimating protein persistence *in vitro *and isolating peptide fragments with potential allergenic epitopes. Therefore the assessment of food allergen requires the use of both digestion and immunology assays as a means to ensure consumer safety to food proteins.

## Competing interests

SS declares that he has no competing interests. RAH is employed by Dow AgroSciences LLC which develops and markets agricultural products, including transgenic crops.

## Authors' contributions

SS and RH collaborated on the conceptualization and preparation of the manuscript equally.
